# Outcomes Following Pars Plana Vitrectomy for Severe Ocular Trauma

**DOI:** 10.18502/jovr.v16i3.9449

**Published:** 2021-07-29

**Authors:** Natalia K. Bober, Neruban Kumaran, Tom H. Williamson

**Affiliations:** ^1^St Thomas’ Hospital, London, UK; ^2^King’s College Medical School, London, UK

**Keywords:** Ocular Trauma, Visual Outcome, Vitrectomy

## Abstract

**Purpose:**

To investigate outcomes and presenting characteristics for subjects undergoing pars plana vitrectomy for ocular trauma.

**Methods:**

Retrospective study of 113 patients who underwent pars plana vitrectomy for severe ocular trauma at [name deleted to maintain the integrity of the review process] between 1999 and 2018. Data were collected on age, gender, initial and final visual acuity (LogMAR), mode of injury, type of injury, number of surgeries performed, follow-up duration, type of tamponade, presence of phthisis, and retinal detachment. The Birmingham Eye Trauma Terminology System (BETTS) was employed.

**Results:**

We identified assault and contusion injuries to be the most common mode and type of ocular injury in our cohort. Furthermore, through follow-up we noted a varied number of operations required by patients presenting with ocular trauma and a statistically significant improvement in visual acuity from 1.73 ( ± 0.86) LogMAR to 1.17 ( ± 1.03; *p* < 0.01) LogMAR. A statistically significant difference in final visual acuity was also noted between BETTS classified type of injury groups (*p* < 0.01). Notably, only 7.3% and 8.2% of patients developed phthisis or a persisting retinal detachment, respectively, during follow-up.

**Conclusion:**

Our study demonstrates that ocular trauma requiring pars plana vitrectomy can require a varied number of operations with a guarded visual prognosis. However, a small percentage will proceed to develop phthisis following intervention.

##  INTRODUCTION

Ocular trauma can cause severe visual impairment with substantial impact on quality of life. Globally, ocular trauma accounts for blindness in approximately 1.6 million people with a further 2.3 million suffering from bilateral low vision and approximately 19 million with unilateral blindness or low vision.^[1]^ Furthermore, it is estimated that there can be around 120,000 of presentations to the Accident and Emergency department due to ocular injuries, annually in the United Kingdom,^[2]^ with hospital admissions estimated to be 8.14 per 100,000 people.^[3]^

Surgical intervention following ocular trauma has two main objectives, firstly to protect ocular integrity and secondly to improve/stabilize vision. The majority of ocular injuries that causes substantial visual loss involve the posterior segment of the eye and as a result these patients often undergo posterior segment (vitreoretinal) surgery.

Pars plana vitrectomy (PPV) allows the reconstruction of the posterior segment of the eye, clear visually significant vitreous opacities, control inflammation, and where appropriate treat retinal detachment. It can also be used to manage posterior segment complications of closed-globe/contusion injuries including retinal detachment, macular hole, and vitreous hemorrhage.^[4]^ PPV was found to have a good prognosis, contributing to significant improvements in visual outcomes following ocular trauma.^[5,6]^ When used as a primary procedure, it resulted in more eyes achieving the final vision of light perception (LP) or better, compared to those that did not undergo the surgery.^[5]^ An important complication of open globe trauma and associated retinal detachment is proliferative vitreoretinopathy, an intraocular scarring process, which can lead to recurrent retinal detachment, associated with poor final visual outcomes.^[7]^

Many groups have investigated independent prognostic factors for ocular trauma. Commonly reported poor prognostic factors include poor initial visual acuity, relative afferent pupillary defect, multiple surgeries, posterior ocular (zone III, > 5 mm posterior to the limbus) injury, vitreous hemorrhage, hyphema, lid laceration, and posterior wound location.^[5,6][8][9][10]^ This was taken further by one group, who developed Ocular Trauma Score (OTS).^[11]^ This score system uses the information of the initial visual acuity, presence of globe rupture, endophthalmitis, perforating injury, retinal detachment, and relative afferent pupillary to estimate the visual outcomes.

Furthermore, given a lack of standardization of classifications and definitions used in ocular trauma studies, the Birmingham Eye Trauma Terminology System (BETTS) was created, introducing a simple and consistent way of classifying ocular trauma, allowing easier and more objective comparison.^[12]^

The epidemiology of eye injuries is known to vary depending on the population studied and is influenced by multiple factors including lifestyle, socioeconomic status, traffic state, and recreational activities.^[13]^

A number of studies have investigated the epidemiology of ocular trauma. Knowledge of specific risk factors associated with ocular injuries in a population can contribute to targeted campaigns that aim to reduce the incidence of ocular trauma in the community.^[14]^ This in turn may involve specifically designed programs to increase the workplace safety and awareness of ocular injuries.^[15]^

Herein, we aim to investigate the frequency of both different modes and types (based on BETTS) of ocular trauma and surgical outcomes of PPV.

##  METHODS

A retrospective analysis of 113 patients who had undergone PPV at St Thomas’ Hospital, London, UK between July 23, 1999 and June 25, 2018 was conducted using the VITREOR database (Microsoft Access, Albuquerque, USA). The study was completed in adherence to the tenets of the Declaration of Helsinki and received appropriate local approvals. All patients underwent PPV for posterior segment complications requiring surgical intervention. Data were collected on age, gender, visual acuity (LogMAR), mode of injury, type of injury, type of tamponade, number of surgeries performed, and time of follow-up. Furthermore, outcomes of phthisis and retinal detachment were noted. Mode of injury was classified into assault, domestic, industrial, road traffic accidents, and sport-related injury. Similarly, the type of ocular injury was categorized as per the BETTS classification, that is, as follows: contusion (without a full-thickness wound to the eye), intraocular foreign body (IOFB), penetrating (entrance wound alone), perforating (entrance and exit wound), and rupture (full thickness wound caused by a blunt trauma).^[12]^ All patients included in the study, including those with ocular contusions, underwent PPV.

All statistical analysis was performed using the SPSS Statistics software package (IBM, New York, USA). The perforating injury group was excluded from analysis due to its comparatively low number. A Chi-square test of independence was used to investigate the difference in frequency of ocular trauma between men and women. The mean preoperative and postoperative visual acuities were compared using a paired *t*-test. A one-way analysis of variance (ANOVA) and a subsequent Tukey's post-hoc test were used to investigate whether there was a difference in the age of presentation between each mode of injury and whether different BETTS classified types of injury were associated with the need for greater or fewer operations and a better or worse final visual acuity. Multiple regression analysis was undertaken to investigate age, gender, initial visual acuity, and type of ocular injury (as defined by the BETTS classification) on final visual acuity.

The study was completed in adherence to the tenets of the Declaration of Helsinki and received appropriate local approvals.

##  RESULTS

### Cohort Demographics

The study included 113 patients, of whom 99 were male and 14 female. Age ranged from 6.2 years to 87.7 years with a mean age of 42.7 years (SD: ± 18.8 years). Longitudinal data were available for 110 patients with follow-up ranging from 1 day to 12.1 years with a mean follow-up of 1.8 years ( ± 2.5).

Men were found to have a statistically significantly higher risk of ocular trauma, contributing to 87.6% of all analyzed cases (*p* < 0.01).

**Figure 1:**
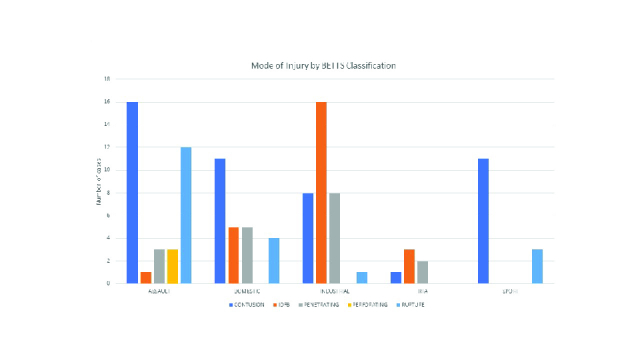
Histograms showing BETTS classified type of injury in each mode of injury. This demonstrates increased frequency of contusion injuries in those suffering an assault and an increased incidence of intraocular foreign bodies in industrial injuries.

In terms of mode of injury, assault (35 cases) and industrial (33 cases) were the most common modes of injury, followed by domestic (25 cases) and sport (14 cases). Least common were road traffic accidents (6 cases). As per the BETTS classification, contusion was the most common type of injury recorded (47 cases), followed by IOFB (25 cases). Notably, all IOFB injuries included posterior segment involvement. As demonstrated in Figure 1, contusion injuries appeared to be most common in those suffering an assault (16 cases) while IOFB were most common in industrial injuries (16 cases). Furthermore, we identified a statistically significant difference in age of presentation between mode of injury groups (*p* < 0.01). Post-hoc analysis revealed a higher mean age at presentation in domestic injuries (54.1 ± 25.0 years) when compared to the sports-related injuries (33.6 ± 19.4 years, *p* < 0.01) and industrial injuries (38.0 ± 12.3 years, *p* < 0.01).

**Figure 2:**
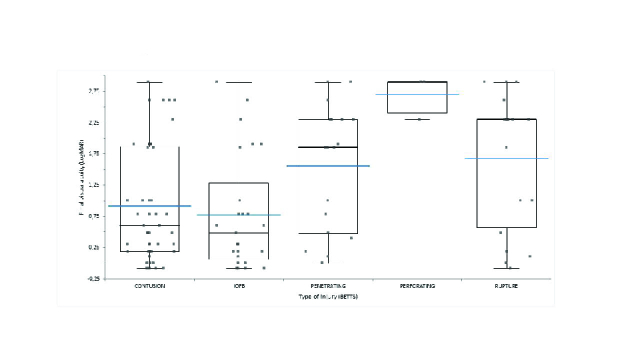
Combined stacked scatterplots and box plots of final visual acuity outcomes in each BETTS classified type of injury. Shown are stacked scatterplots and box plots for contusion injuries (45 eyes), intraocular foreign bodies (25 eyes), penetrating injuries (18 eyes), perforating injuries (3 eyes), and globe rupture (19 eyes). Box plots with box spanning interquartile range with maximum and minimum values (excluding outliers) are provided. Black and blue lines represent median and mean, respectively.

### Surgical Outcomes

The number of operations required varied from one to five with a mean number of 1.8 ( ± 1.0) operations performed. We identified a statistically significant difference in the number of operations required between the BETTS classified types of injury (*p* = 0.02). The statistically significant difference was found when comparing the number of operations required for contusion injury, mean 1.51 ( ± 0.91) with IOFB injury, mean 2.28 ( ± 1.24, *p* = 0.02), which are also the groups with respectively the smallest and biggest average numbers of surgeries performed per BETTS group.

In our cohort, on follow-up, an improvement from presenting visual acuity was noted in 73 patients (66%); however, 25 patients (23%) were found to have a deterioration in visual acuity, while 12 patients (11%) were found to have no change.

Furthermore, the mean preoperative visual acuity in our cohort was 1.73 ( ± 0.86) LogMAR which was noted to improve postoperatively to a mean of 1.17 ( ± 1.03; *p* < 0.01) LogMAR.

Overall, all BETTS groups were noted to have improvement in final visual acuity, with the only exception of perforating group [Table 2]. Interestingly, as demonstrated in Figure 2, a statistically significant difference in final visual acuity was noted between BETTS classified type of injury groups (*p* < 0.01). Post-hoc analysis revealed that final visual acuity was statistically significantly better in the contusion group 0.92 ( ± 0.91) LogMAR compared to the rupture group 1.68 ( ± 1.08, *p* = 0.02) LogMAR. Visual acuity was also found to be better in the IOFB group 0.78 ( ± 0.92) LogMAR when compared to the penetrating group 1.55 ( ± 1.00, *p* = 0.05) LogMAR and rupture group (*p* = 0.01).

**Table 1:** Demographics showing number of patients and age (mean and standard deviation) for each mode of injury


**Mode of injury**	**Number of patients**	**Mean age**	**Standard deviation**
Assault	35	41.3	15.0
Domestic	25	54.1	25.0
Industrial	33	38.0	12.3
Road traffic accident	6	49.5	19.0
Sport	14	33.6	19.4
**Total**	**113**	**42.7**	**18.8**

**Table 2:** Initial pre-op and final post-op visual acuity with overall visual acuity change in different BETTS groups (mean and standard deviation)[LogMAR]


**Cause of PPV**	**Pre-op VA (Mean ± SD) [LogMAR]**	**Post-op VA (Mean ± SD) [LogMAR]**	**VA change**
Contusion	1.52 ± 0.85	0.92 ± 0.91	0.60 ± 0.19
IOFB	1.40 ± 1.01	0.78 ± 0.92	0.62 ± 0.27
Penetrating	1.95 ± 0.67	1.55 ± 1.00	0.40 ± 0.28
Perforating	2.43 ± 0.29	2.70 ± 0.35	–0.27 ± 0.26
Rupture	2.03 ± 0.59	1.68 ± 1.08	0.35 ± 0.28
PPV, pars plana vitrectomy; IOFB, intraocular foreign body; VA, visual acuity; LogMAR, logarithm minimum angle of resolution; SD, standard deviation			

**Table 3:** Frequency of different types of tamponade use in BETTS groups with overall final visual acuity[LogMAR]


**Cause of PPV**	**Percentage requiring gas tamponade**	**Percentage requiring oil tamponade**	**Percentage requiring no tamponade**	**Mean final visual acuity [LogMAR]**
Contusion	37.8% (*n* = 17)	28.9% (*n* = 13)	33.3% (*n* = 15)	0.92 ± 0.91
IOFB	28.0% (*n* = 7)	52.0% (*n* = 13)	20.0% (*n* = 5)	0.78 ± 0.92
Penetrating	33.3% (*n* = 6)	50.0% (*n* = 9)	16.7% (*n* = 3)	1.55 ± 1.00
Perforating	0%	100% (*n* = 3)	0%	2.70 ± 0.35
Rupture	10.5% (*n* = 2)	63.2% (*n* = 12)	26.3% (*n* = 5)	1.68 ± 1.08
Overall	30.9% (*n* = 32)	43.6% (*n* = 50)	25.5% (*n* = 28)	
**Mean final visual acuity**	0.81 ± 0.90	1.70 ± 0.97	0.63 ± 0.84	
IOFB, intraocular foreign body; LogMAR, logarithm minimum angle of resolution				

Patients who underwent PPV in our study required gas, oil, or no tamponade. Gases used include SF6, C2F6, C3F8, and Air. Oil tamponade was found to be most commonly used in all BETTS groups 43.6% (*n* = 48) except contusion, followed by gas tamponade 30.9% (*n* = 34). PPV without tamponade was least commonly performed 25.5% (*n* = 28). Best final visual acuity was found in a group with no tamponade 0.63 ( ± 0.84) LogMAR, followed by gas tamponade 0.81 ( ± 0.90) LogMAR. As expected, oil tamponade was associated with the worst final visual acuity 1.70 ( ± 0.97) LogMAR [Table 3].

In our cohort, eight patients (7.3%) developed phthisis during their follow-up, with associated poor visual outcomes (with documented visual acuity ranging from hand movement to no LP). Out of all eight patients, three noted no change and five had visual deterioration. Furthermore, of the phthisis cases, four patients presented with ocular rupture – three with perforating injury and one in IOFB injury, as per the BETTS classification system. Retinal detachment persisted in nine patients (8.2%) with results of visual acuity improvement in one patient, no change in three patients, and deterioration in five patients.

A multiple regression analysis was run to predict final VA from age, gender, initial visual acuity, and type of ocular injury. Presenting VA (*p* < 0.01) and type of ocular injury (*p* = 0.046) were identified as statistically significant in predicting final VA.

##  DISCUSSION

Ocular trauma is known to be an important cause of visual loss with varying groups investigating ocular trauma in different countries in different settings.^[5,6][16]^ In this study, we investigated both cross-sectional and longitudinal data in a large cohort of patients presenting with ocular trauma to a large tertiary referral center in London, UK.

We identified men to have a statistically significantly increased risk of ocular trauma, in keeping with multiple other studies.^[3,5,10,16]^ Furthermore, we identified a novel finding of assault as the commonest cause of ocular trauma in our cohort. Assault has previously been described as a risk factor for poor visual outcomes.^[17]^ While other groups have identified home^[3]^ and workplace^[6]^ as common locations, little has been known about the nature of ocular trauma outside of a conflict setting.^[10]^ Similarly, we also identified sports-related ocular injury to be more common in younger patients. This further emphasizes the importance of education on eye protection during sporting activities.

Through investigating outcomes using the BETTS classification,^[12]^ we identified contusion injuries to be the most common, and furthermore required the least number of operations, achieving a statistically significantly better final visual acuity, when compared to the rupture group. Contusion injuries by definition do not have a full-thickness ocular wound, and as such they would be expected to have a better prognosis.

Furthermore, we identified a guarded improvement in visual acuity following PPV for ocular trauma with improvement or stability of visual acuity noted in 66% of patients and an improvement from 1.73 ( ± 0.86) LogMAR to 1.17 ( ± 1.03; *p* < 0.01) LogMAR. The improvement was noted in all BETTS groups with the only exception of perforating group. While this does demonstrate a statistically significant improvement in visual acuity, it also highlights the guarded prognosis of final visual acuity for PPV following ocular trauma.

Notably, other groups have demonstrated similar improvements following PPV, albeit with different cohorts to ours. Mansouri et al described an improvement from 2.36 ( ± 0.72) LogMAR to 1.50 ( ± 1.14) LogMAR following PPV in a smaller cohort (*n* = 90) in Iran.^[6]^ Furthermore, Guven et al described a statistically significant improvement in 337 out of their cohort of 633 patients who underwent PPV following ocular trauma.^[10]^ However, it should also be noted that a large proportion (48.2%) of patients in this cohort had terror-related open globe injury, making it difficult to compare their findings with those of this study. Interestingly, Xia et al also describe an improvement in visual acuity from 2.20 ( ± 0.63) LogMAR to 1.87 ( ± 0.60) LogMAR in a longitudinal American cohort of 96 eyes, similar to ours.^[16]^

Overall, oil tamponade was the most commonly chosen agent and that group had worst final visual acuity 1.70 ( ± 0.97) LogMAR; however, the reason for this was most likely the type and severity of ocular injury, with the need for a longer acting tamponade. Therefore, in our study, type of tamponade cannot be used as a prognostic factor on its own. Similarly, Jiang et al concluded that use of silicone oil did not affect the visual acuity prognosis but the severity of presentation, in that case traumatic endophthalmitis, was the main factor determining the appropriate choice of treatment.^[18]^ Vaziri et al concluded that the type of tamponade used should be individualized according to the characteristics of the patient and medical presentation.^[19]^

Additionally, only 8 and 9 patients out of 110 patients followed-up developed phthisis and a persisting retinal detachment, respectively, at the final visit. Furthermore, there was a wide variation of one to five surgeries required for patients with ocular trauma in our cohort, with a mean of 1.8 ( ± 1.0) operations similar to other cohorts.^[16]^ Furthermore, we identified that presenting VA (*p* < 0.01) and type of ocular injury (*p* = 0.05) as prognostic indicators for final VA again suggesting that those with less severe injuries on presentation have a better prognosis. Indeed, initial VA was shown as an important prognostic factors in multiple previous studies.^[5,10,20,21]^

There are, however, limitations to our study. We were unable to investigate the presence of endophthalmitis, presence of afferent pupillary defect and zone of injury (as previously explored by the OTS), and the impact of this on surgical outcome. Secondly, our study does not involve information on the timing of the PPV. Currently, there are no clear guidelines concerning the optimal timing of surgery. According to Ryan et al and Brinton et al, patients that underwent vitrectomy within first 14 days after presentation seemed to have better visual outcomes than those who had to wait longer.^[22,23]^ However, others argue the timing of vitrectomy is not related to visual outcomes.^[6,24]^ For that reason it is difficult to estimate the effect that timing of the PPV may have. Additionally, in the contusion group we were unable to investigate the specific indication for intervention, and in the IOFB group while all IOFBs involved the posterior segment, we were unable to investigate the location within the posterior pole. What is more, the retrospective nature of the study can introduce bias due to variability in reporting clinical findings. Furthermore, as a single-center study, it is difficult to use our findings to inform practices in other countries.

In summary, we present a study of prognostic factors and outcomes of vitreoretinal surgery in ocular trauma. We demonstrate a varied number of surgeries required following ocular trauma, visual acuity improvement following PPV but with a very guarded prognosis and, as expected, that patients without a full-thickness laceration have the best prognosis.

##  Financial Support and Sponsorship

Nil.

##  Conflicts of Interest

There are no conflicts of interest.
